# In Vitro Evaluation of the Antiviral Activity of Methylene Blue Alone or in Combination against SARS-CoV-2

**DOI:** 10.3390/jcm10143007

**Published:** 2021-07-06

**Authors:** Mathieu Gendrot, Priscilla Jardot, Océane Delandre, Manon Boxberger, Julien Andreani, Isabelle Duflot, Marion Le Bideau, Joel Mosnier, Isabelle Fonta, Sébastien Hutter, Bernard La Scola, Bruno Pradines

**Affiliations:** 1 Unité Parasitologie et Entomologie, Département Microbiologie et Maladies Infectieuses, Institut de Recherche Biomédicale des Armées, 13005 Marseille, France; ma.gendrot@laposte.net (M.G.); o.delandre@gmail.com (O.D.); joelmosnier@orange.fr (J.M.); isabelle.fonta.09@gmail.com (I.F.); 2 Aix Marseille Univ, IRD, SSA, AP-HM, VITROME, 13005 Marseille, France; sebastien.hutter@univ-amu.fr; 3 IHU Méditerranée Infection, 13005 Marseille, France; Priscilla.JARDOT@univ-amu.fr (P.J.); manon.boxberger@hotmail.fr (M.B.); miaguiabidou@gmail.com (J.A.); isabelle.duflot@etu.univ-amu.fr (I.D.); Marion.LE-BIDEAU@ap-hm.fr (M.L.B.); bernard.la-scola@univ-amu.fr (B.L.S.); 4 Aix Marseille Univ, IRD, AP-HM, MEPHI, 13005 Marseille, France; 5 Centre National de Référence du Paludisme, 13005 Marseille, France

**Keywords:** COVID-19, SARS-CoV-2, antiviral, in vitro, methylene blue, antimalarial drug

## Abstract

A new severe acute respiratory syndrome coronavirus (SARS-CoV-2) causing coronavirus diseases 2019 (COVID-19), which emerged in Wuhan, China in December 2019, has spread worldwide. Currently, very few treatments are officially recommended against SARS-CoV-2. Identifying effective, low-cost antiviral drugs with limited side effects that are affordable immediately is urgently needed. Methylene blue, a synthesized thiazine dye, may be a potential antiviral drug. Antiviral activity of methylene blue used alone or in combination with several antimalarial drugs or remdesivir was assessed against infected Vero E6 cells infected with two clinically isolated SARS-CoV-2 strains (IHUMI-3 and IHUMI-6). Effects both on viral entry in the cell and on post-entry were also investigated. After 48 h post-infection, the viral replication was estimated by RT-PCR. The median effective concentration (EC_50_) and 90% effective concentration (EC_90_) of methylene blue against IHUMI-3 were 0.41 ± 0.34 µM and 1.85 ± 1.41 µM, respectively; 1.06 ± 0.46 µM and 5.68 ± 1.83 µM against IHUMI-6. Methylene blue interacted at both entry and post-entry stages of SARS-CoV-2 infection in Vero E6 cells as retrieved for hydroxychloroquine. The effects of methylene blue were additive with those of quinine, mefloquine and pyronaridine. The combinations of methylene blue with chloroquine, hydroxychloroquine, desethylamodiaquine, piperaquine, lumefantrine, ferroquine, dihydroartemisinin and remdesivir were antagonist. These results support the potential interest of methylene blue to treat COVID-19.

## 1. Introduction

In December 2019, a new coronavirus called severe acute respiratory syndrome coronavirus (SARS-CoV-2) responsible for coronavirus diseases 2019 (COVID-19) was first detected in Wuhan, China, before spreading all over the world [1]. SARS-CoV-2 belongs to the *Betacoronaviruses* with similarities with viruses detected in bats [2]. SARS-CoV-2 caused a wide range of symptoms from asymptomatic to fatal respiratory tract infections [2,3,4]. The common symptoms of COVID-19 are fever with dry cough, dyspnea, headaches, myalgia, intense fatigue with gastrointestinal symptoms including vomiting, abdominal pain, loss of appetite and diarrhea [2,3,4]. Less common symptoms including hyposmia, anosmia, ageusia, maculopapular rash or urticarious lesions are observed [4]. Severe cases are associated with uncontrolled increased lung inflammatory response called cytokine storm syndrome. An interleukin release of IL-6, IL-1, IL-2, IL-10, IL-12 and IL-18 associated with tumour necrosis factor alpha (TNF-α) and other inflammatory mediators (IP-10, MCP-1, MIP-1α) are associated with the severity of pulmonary inflammation and extensive lung damages, likely leading to death [3,5]. Currently, very few treatments are officially recommended against SARS-CoV-2. Evaluation of repurposing of existing approved drugs is an efficient, low-cost approach to identify therapeutic against SARS-CoV-2. Several compounds have been already evaluated at least in vitro, including antimalarial drugs (chloroquine, mefloquine, quinine, pyronaridine, piperaquine, lumefantrine, artemisinin) [6,7,8,9], antibiotics (azithromycin, doxycycline) [10,11], antiparasitic drugs (ivermectin) [12] or antiviral agents (remdesivir, ritonavir, lopinavir, favipiravir) [8,9,13,14].

Methylene blue, a synthesized thiazine dye, is able to inactivate viruses, including Zika, yellow fever, dengue, chikungunya, Ebola viruses and Middle East respiratory syndrome coronavirus in plasma when illuminated with visible light [15,16,17,18]. Methylene blue was also shown to exert in vitro and in vivo antimicrobial effects without photoactivation, and more particularly against *Plasmodium spp.* [19,20,21,22,23]. The repurposed methylene blue could be a potent candidate in the treatment of COVID-19 [24]. SARS-CoV-2 in plasma or in infected Vero E6 cells was inactivated by photoactivation [25,26,27]. Moreover, methylene blue was found to inhibit SARS-CoV-2 in vitro at concentrations achievable after oral or intravenous administration [25,28].

The aim of this study was to confirm the antiviral activity of methylene blue against SARS-CoV-2, to investigate its effects on viral entry in the cell and on post-entry and its activity in combination with other potential drugs.

## 2. Materials and Methods

### 2.1. Drugs, Virus and Cells

Methylene blue (methylthioninium chloride; Proveblue^®^) was provided by Provepharm SAS (Marseille, France). Hydroxychloroquine sulfate (Sigma Aldrich, St Quentin Fallavier, France) and remdesivir (Apollo Scientific, Manchester, UK) were used as comparators. Stock solutions of methylene blue and hydroxychloroquine were prepared in water and remdesivir in DMSO/water 10%. All the stock solutions were then diluted in Minimum Essential Media (MEM, Gibco, ThermoFischer, Waltham, MA, USA) in order to have 7 final concentrations ranging from 0.1 µM to 100 µM. Two clinically-isolated SARS-CoV-2 strains (IHUMI-3 and IHUMI-6), collected in hospitalized patients during the first COVID-19 outbreak in March 2020 in Marseille [29], were maintained in production in Vero E6 cells (American type culture collection ATCC^®^ CRL-1586™) in MEM with 4% of fetal bovine serum and 1% of glutamine (complete medium). Vero E6 cells are one of the most used cells for the culture of SARS-CoV-2 due to the presence of high expression of angiotensin converting enzyme 2 (ACE2) receptors, essential for SARS-CoV-2 cellular entry [30,31]. Vero E6 cells were found to be relevant for antiviral drug screening models [31,32].

### 2.2. Antiviral Activity Assay

Briefly, 96-well plates were prepared with 5 × 10^5^ cells/mL of Vero E6 (200 µL per well), as previously described [10]. The different concentrations of methylene blue without photoactivation, hydroxychloroquine or remdesivir were added 4 h before infection. The replication of IHUMI-3 or IHUMI-6 strains in Vero E6 cells at an MOI of 0.01 was estimated 48 h after infection by RT-PCR using the Superscript III platinum one step with Rox kit (Invitrogen) after extraction with the BioExtract SuperBall kit (Biosellal, Dardilly, France). The primers used were previously described [33]. EC_50_ (median effective concentration) and EC_90_ (90% effective concentration) were estimated through nonlinear regression by using the R software (ICEstimator version 1.2). EC_50_ and EC_90_ values resulted in the mean of 6 to 12 independent experimentations.

### 2.3. Determination of the Inhibition Stage

Effects of methylene blue, hydroxychloroquine or remdesivir on entry and post-entry of SARS-CoV-2 were evaluated at a concentration of 10 µM. For “full-time” treatment, Vero E6 cells were infected with the IHUMI-3 strain for 48 h after pre-incubation of the cells with one of the three drugs for 4 h. For “entry” treatment, the cells were infected for 2 h after pre-incubation for 4 h and then the virus–drug mixture was replaced with fresh medium maintained for 46 h. For “post-entry” treatment, the cells were infected for 2 h and then incubated with drug for 46 h. The percentage of inhibition of SARS-CoV-2 replication by 10 µM of drug was estimated for each drug concentration as following: (mean CT_drug concentration_ − mean CT_control 0%_)/(mean CT_control 100%_ − mean CT_control 0%_) × 100. The result was the mean of 6 to 9 independent experiments.

### 2.4. Antiviral Activity of Drug Combinations

The antiviral activity of two concentrations of methylene blue (0.1 and 0.5 µM) was evaluated alone or in combination with four fixed concentrations of chloroquine (0.5, 1, 5 and 10 µM), hydroxychloroquine (0.5, 1, 5 and 10 µM), quinine (1, 5, 10 and 25 µM), mefloquine (0.5, 1, 5 and 10 µM), pyronaridine (0.1, 0.5, 1 and 5 µM), ferroquine (0.5, 1, 5 and 10 µM), desethylamodiaquine (0.1, 0.5, 1 and 10 µM), lumefantrine (5, 10, 25 and 50 µM), piperaquine (5, 10, 25 and 50 µM), dihydroartemisinin (5, 10, 25 and 50 µM) and remdesivir (0.05, 0.1, 0.5 and 1 µM) against the SARS-CoV-2 IHUMI-3 strain for 48 h. The percentage of inhibition of SARS-CoV-2 replication by methylene blue alone or in combination was estimated for each drug association as following: (mean CT_drug association_ − mean CT_control 0%_)/(mean CT_control 100%_ − mean CT_control 0%_) × 100. The result was the mean of 9 to 13 independent experiments.

## 3. Results

**Figure 1:**
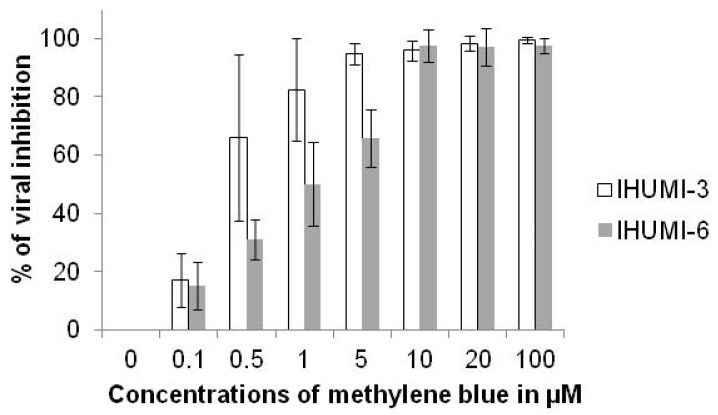
Anti-SARS-CoV-2 activity of methylene blue in % of antiviral inhibition on IHUMI-3 (mean of 12 independent experiments) and IHUMI-6 (mean of 6 independent experiments) clinically-isolated strains (error bar represents standard deviation).

The antiviral activity of methylene blue against the clinically-isolated SARS-CoV-2 strains IHUMI-3 and IHUMI-6 was concentration-dependent (Figure 1).

The median effective concentration (EC_50_) and 90% effective concentration (EC_90_) of methylene blue against IHUMI-3 were 0.41 ± 0.34 µM and 1.85 ± 1.41 µM (n = 12), respectively; 1.06 ± 0.46 µM and 5.68 ± 1.83 µM against IHUMI-6 (n = 6). The difference between EC_50_ against the two was significant (*p* = 0.015, Welch two sample *t*-test).

In comparison, EC_50_ and EC_90_ of remdesivir against IHUMI-6 were 1.00 ± 0.41 µM and 3.2 ± 2.9 µM, respectively (n = 6). There was no significant difference between methylene blue and remdesivir EC_50_ or EC_90_ (*p* = 0.786 and *p* = 0.113, Welch two sample *t*-test).

EC_50_ and EC_90_ of hydroxychloroquine against IHUMI-6 were 6.25 ± 2.20 µM and 12.32 ± 2.82 µM, respectively (n = 6). Methylene blue was significantly more effective than hydroxychloroquine against IHUMI-6 (*p* = 0.005 for EC_50_ and *p* = 0.003 for EC_90_; Welch two sample *t*-test).

**Figure 2:**
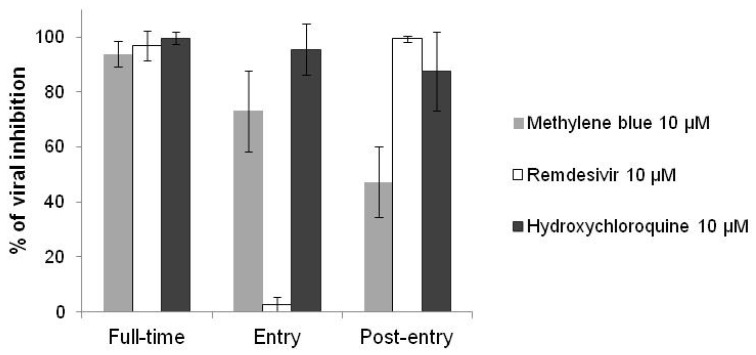
Antiviral activities of methylene blue, hydroxychloroquine and remdesivir at 10 µM against the SARS-CoV-2 IHUMI-3 strain in vitro. For “full-time” treatment, Vero E6 cells were infected with the IHUMI-3 strain for 48 h after pre-incubation of the cells with one of the three drugs for 4 h. For “entry” treatment, the cells were infected for 2 h after pre-incubation for 4 h and then the virus–drug mixture was replaced with fresh medium maintained for 46 h. For “post-entry” treatment, the cells were infected for 2 h and then incubated with drug for 46 h. Error bars represent standard deviation of 6 to 9 independent experiments.

Methylene blue interacted at both entry and post-entry stages of SARS-CoV-2 infection in Vero E6 cells, as hydroxychloroquine did (Figure 2). Contrariwise, remdesivir, which is an antiviral drug, interacted only at post-entry stage.

**Figure 3:**
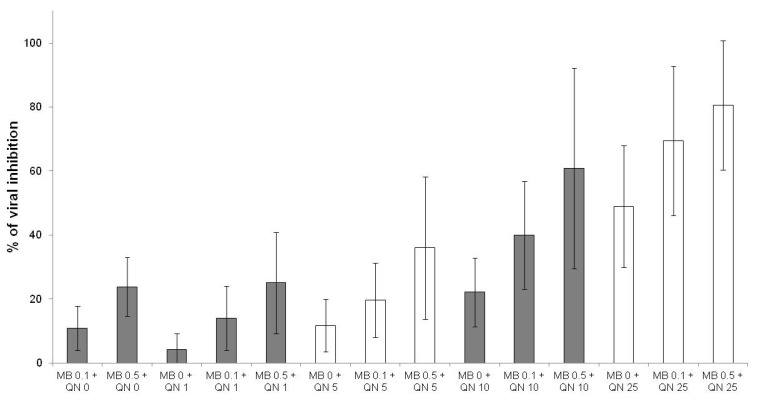
Antiviral activities of methylene blue (MB) at 0.1 and 0.5 µM in combination with quinine (QN) at 1, 5, 10 and 25 µM (error bars represent standard deviation of 13 independent experiments).

**Figure 4:**
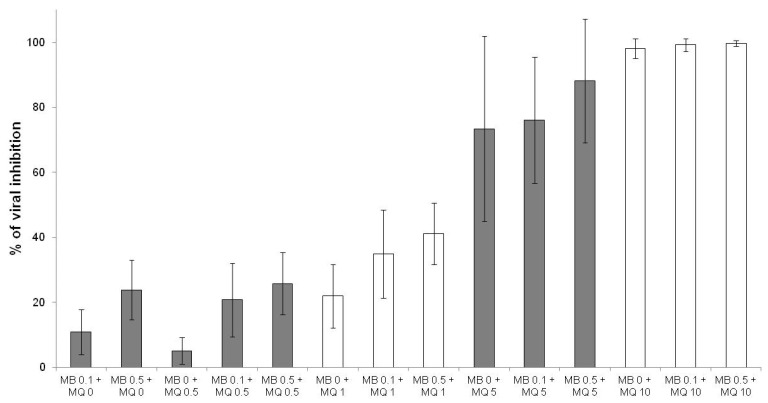
Antiviral activities of methylene blue (MB) at 0.1 and 0.5 µM in combination with mefloquine (MQ) at 0.5, 1, 5 and 10 µM (error bars represent standard deviation of 13 independent experiments).

**Figure 5:**
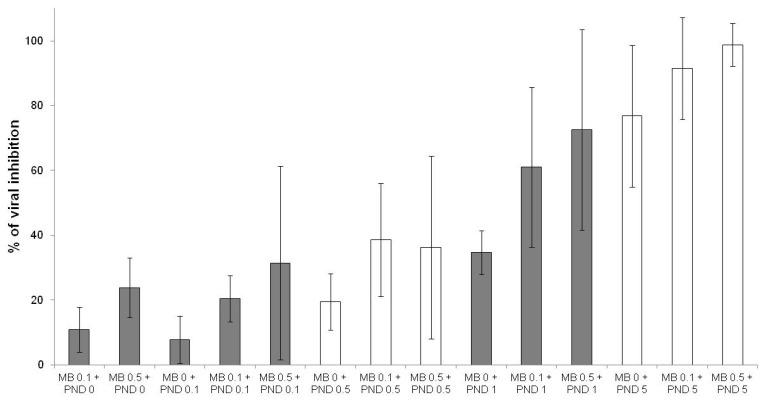
Antiviral activities of methylene blue (MB) at 0.1 and 0.5 µM in combination with pyronaridine (PND) at 0.1, 0.5, 1 and 5 µM (error bars represent standard deviation of 9 independent experiments).

The effects of methylene blue were additive with those of quinine (Figure 3), mefloquine (Figure 4) and pyronaridine (Figure 5).

**Figure 6:**
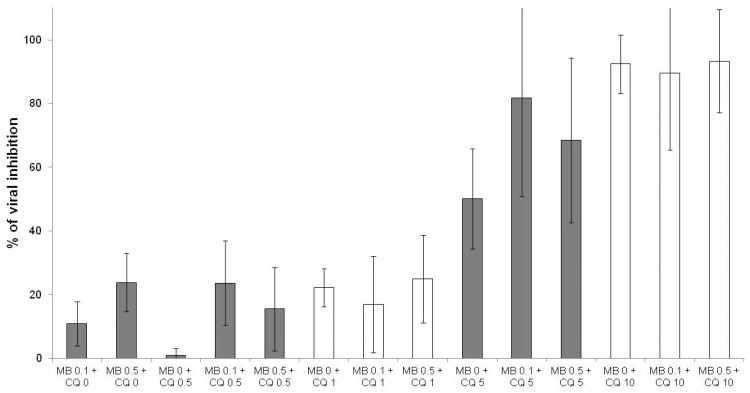
Antiviral activities of methylene blue (MB) at 0.1 and 0.5 µM in combination with chloroquine (CQ) at 0.5, 1, 5 and 10 µM (error bars represent standard deviation of 9 independent experiments).

The combinations of methylene blue with chloroquine (Figure 6), hydroxychloroquine, desethylamodiaquine, piperaquine, lumefantrine, ferroquine, dihydroartemisinin and remdesivir (data not shown) were antagonist.

## 4. Discussion

Our data confirmed the in vitro activity of methylene blue at very low-micromolar range with EC_50_ between 0.41 and 1.06 µM and EC_90_ between 1.85 ± 1.41 µM and 5.68 ± 1.83 µM against two strains of SARS-CoV-2 IHUMI-3 and IHUMI-6 [28,34,35]. The reduction in the viral replication is not due to methylene blue toxicity against Vero E6 cells. The 50% cytotoxic concentration (CC_50_) was previously evaluated (CC_50_ > 100 µM) [28]. According to this previous CC_50_, the selectivity index (SI) for methylene blue was above 100. Methylene blue was effective as antiviral remdesivir against IHUMI-6 strain and more effective than hydroxychloroquine in vitro. These effective concentrations are compatible with blood concentrations after usual oral intake or intravenous injection of methylene blue [36,37,38]. An oral uptake of 325 mg of methylene blue led to a C_max_ (maximum blood concentration) value of 0.97 µg/mL (around 3 µM) [36] and a dose of 2 mg/kg intravenous showed a C_max_ of 2.917 µg/mL (around 10 µM) [37]. In another study, blood concentrations of 6–7 µM were obtained after three oral daily doses of 69 mg (207 mg/day) [38]. Methylene blue EC_50_ and EC_90_ are coherent with human blood concentrations after usual uptake of methylene blue. Moreover, methylene blue is accumulated in lungs tissue. The absorption of methylene blue in lungs was around 3 to 5% of injected drug per g of tissue after a single intravenous injection of methylene blue in mice [39].

Methylene blue could be associated with antimalarial drugs such as quinine, mefloquine or pyronaridine to improve its antiviral activity. Mefloquine concentrations are 10 times higher in the lung than in the blood (a concentration which can go up to 180 mg/kg in the lung) [40]. A single oral dose of 2 mg (10 mg/kg) of pyronaridine in rats led to a blood C_max_ of 223 ng/mL and a lung C_max_ of 36.4 µg/g of tissue (165 more concentrated) [41]. In rat, after intravenous dose of 10 mg/kg of quinine, the observed concentration lung/blood ratio was at 246 [42]. These three drugs accumulate in lungs and could be potent partners for methylene blue for COVD-19 treatment.

Methylene blue interacted at both entry and post-entry stages of SARS-CoV-2 infection in Vero E6 cells. The inhibition of the viral entry is consistent with the results interaction between the spike protein (S) and the angiotensin converting enzyme 2 (ACE2) via its receptor binding domain (RBD), binding required for SARS-CoV-2 cell entry. Methylene blue inhibits the binding of SARS-CoV-2 spike S protein to ACE2 at micromolar range [35]. Moreover, the inhibition of both entry and viral replication after SARS-CoV-2 entry is coherent with 3D modelling approaches. Docking analysis showed that methylene blue could bind both the spike protein S of SARS-CoV-2, but lesser than hydroxychloroquine, and the main protease (M or Mpro), but lesser than remdesivir [43]. The main protein, also called 3C-like protease, is crucial in SARS-CoV-2 replication by leading to the formation of non-structural proteins (NSPs) [44]. SARS-CoV-2 needs the transmembrane protease serine 2 (TMPRSS2) for activating the spike S protein [5]. The spike S protein, TMPRSS2 and Mpro are promising anti-SARS-CoV-2 targets for enzymatic inhibitors [5,45]. Moreover, SARS-CoV-2 3D comparative modelling analyses lead to predict interactions with spike protein S and human ACE2 and to design neutralizing antibodies for blocking this binding as a new therapeutic strategy [46,47].

Besides its antiviral activity, methylene blue is reduced into leukomethylene blue which reduces the methemoglobin to hemoglobin. Methylene blue could reduce hypoxia, one of the main complications in COVID-19 patients, by decreasing methemoglobin. Moreover, methylene blue decreases inflammation and oxidative stress [48,49]. Pro-inflammatory cytokines and nitric oxide were considerably increased in the cytokine storm due to COVID-19 [50].

These results support additional in vivo studies in animal experimental models to confirm methylene blue anti-SARS-CoV-2 activity. The probable use of methylene blue to treat COVID-19 needs to be established by prospective comparative clinical studies. Methylene blue has been assessed in combination with vitamin C and N-acetyl cysteine in severe COVID-19 [51,52]. The addition of methylene blue to standard of care treatment significantly improved respiratory distress, hospital stay and mortality rate in severe patients with confirmed COVID-19 [53].

## 5. Conclusions

Methylene blue, an FDA-approved drug for methemoglobinemia treatment, showed potent in vitro anti-SARS-CoV-2 at micromolar range and potentiation in combination with antimalarial drugs, including quinine, mefloquine or pyronaridine. Methylene blue acted at both entry and post-entry (replication) of SARS-CoV-2 in Vero E6 cells. Methylene blue needs additional in vivo evaluation in animal models and then in human to confirm its antiviral effects.

## Data Availability

The data presented in this study are available on request from the corresponding author.
